# Analysis of comprehensive biomolecules in critically ill patients via bioinformatics technologies

**DOI:** 10.1002/ams2.944

**Published:** 2024-04-08

**Authors:** Hisatake Matsumoto, Hiroshi Ogura, Jun Oda

**Affiliations:** ^1^ Department of Traumatology and Acute Critical Medicine Osaka University Graduate School of Medicine Suita Osaka Japan

**Keywords:** heterogeneity, omics, personalized medicine, subtype, translational research

## Abstract

Each patient with a critical illness such as sepsis and severe trauma has a different genetic background, comorbidities, age, and sex. Moreover, pathophysiology changes dynamically over time even in the same patient. Therefore, individualized treatment is necessary to account for heterogeneity in patient backgrounds. Recently, the analysis of comprehensive biomolecular information using clinical specimens has revealed novel molecular pathological classifications called subtypes. In addition, comprehensive biomolecular information using clinical specimens has enabled reverse translational research, which is a data‐driven approach to the identification of drug target molecules. The development of these methods is expected to visualize the heterogeneity of patient backgrounds and lead to personalized therapy.

## Graphical abstract

The critically ill patients’ background is heterogeneous and requires individually optimized treatment. Advances in comprehensive biomolecular information measurement and bioinformatics technologies have made it possible to analyze molecular pathogenesis in detail and this enabled reverse translational research for drug discovery.

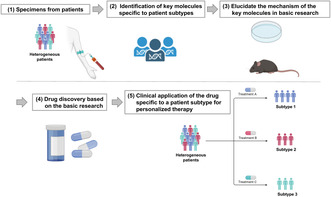

## INTRODUCTION

In critical illness including sepsis and severe trauma, patients exhibit diverse genetic backgrounds, comorbidities, ages, and sexes. Additionally, the pathophysiology can change dynamically over time even in the same patient. Therefore, individualized treatment is crucial to address the heterogeneity in patient backgrounds.

Recent technological advancements have facilitated accurate analysis of the precise evaluation of comprehensive biomolecular information (omics information) including comprehensive DNA, RNA, and protein profiles. Concurrently, the progression in bioinformatics technology has enriched the analysis of omics data, unveiling new pathological insights and elucidating drug target molecules. These advancements have allowed the analysis of comprehensive biomolecular information using clinical specimens, unveiling new molecular pathological classifications called subtypes.

Comprehensive biomolecular information using clinical specimens has allowed reverse translational research, which is a data‐driven methodology that refers to an approach relying extensively on analyzing data based on the clinical specimens to identify drug target molecules, rather than starting with a hypothesis or theory. These advancements are expected to elucidate the heterogeneity of patient backgrounds, leading to personalized therapy. We review the latest findings and discuss current challenges and future prospects.

## HETEROGENEITY ASSESSMENT OF CRITICAL ILLNESS: DAWN OF THE PRECISION ERA

**FIGURE 1:**
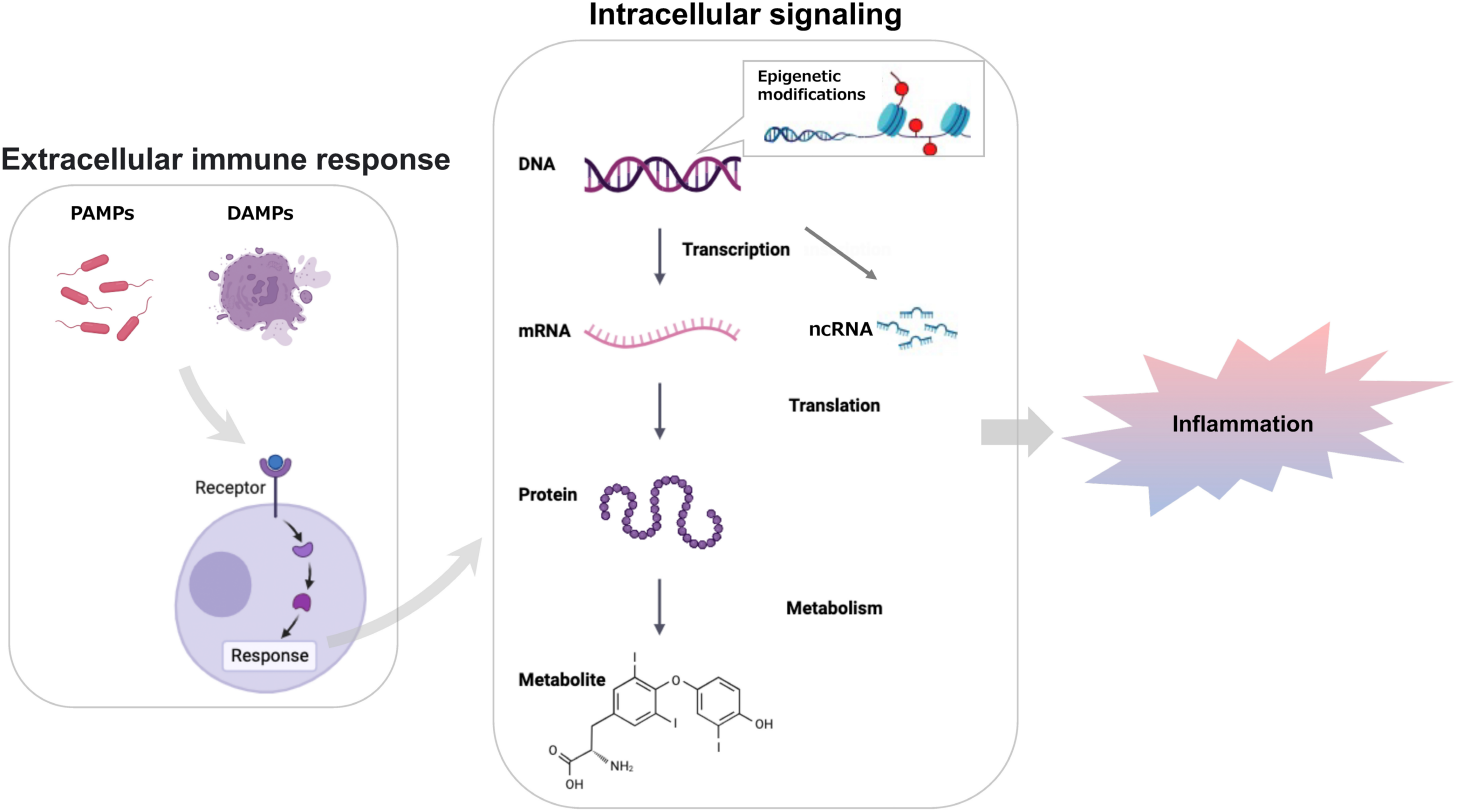
Immune and inflammatory responses in critical illness. As an extracellular immune response, pathogen‐associated molecular patterns (PAMPs) and damage‐associated molecular proteins (DMAPs) bind to immunocompetent cells. Activated intracellular transcription factors bind to nuclear DNA and transcribe RNA (mRNA: messenger RNA) and nonprotein‐coding RNA (ncRNA: noncoding RNA). Transcription itself is also altered by epigenetics, in which certain genes are modified by invasion. Proteins are translated from transcribed mRNA, and inflammatory reactions proceed in both intracellular and extracellular immune responses.

**FIGURE 2:**
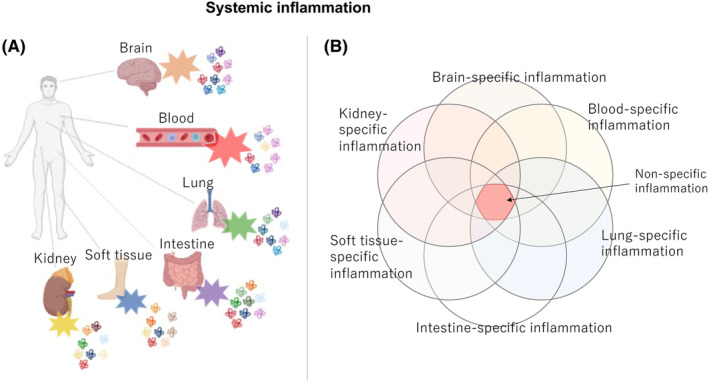
Systemic inflammation in critical illness. Inflammation spills over into various tissues and organs throughout the body, including the vascular endothelium. Various cascades are associated with each type of inflammation, including immune reactions (e.g., cytokines with specific or common characteristics, coagulation reactions, regenerative reactions, and metabolic reactions). (A) Specific types of inflammation are observed in individual organs, whereas (B) non‐specific inflammation can be found throughout all organs.

Critical illness (e.g., sepsis, severe burns, severe trauma, other bioinvasive conditions) induces a systemic inflammatory response. Pathogen‐associated molecular patterns (PAMPs), such as viral and bacterial pathogens, and damage‐associated molecular patterns (DMAPs) associated with cellular injury bind as ligands to pattern recognition receptors on immunocompetent cells (e.g., monocytes) and induce inflammation. Activated intracellular transcription factors bind to DNA in the nucleus, resulting in the transcription of messenger RNA (mRNA) and nonprotein‐coding RNA (ncRNA). Recently, ncRNAs that do not code for proteins have been found to be involved in inflammatory responses. It has also been reported that certain genes are modified by invasion (i.e., epigenetics) and that epigenetics affects transcription. The transcribed mRNAs translate proteins, which lead to a progressive inflammatory response **(**Figure 1 **)** that can lead to fatal disseminated intravascular coagulation syndrome and multiorgan damage **(**Figure 2 **)**.^1^, ^2^

Practice involving critical illness can be divided into three major eras.^3^ The first was the “basic era” (1955–1980s), during which continuous monitoring of mechanical ventilation and physiologic parameters was introduced and nurse‐to‐patient ratios improved. The second was the “era of acceleration” (1980s to approximately 2020), which saw an improved understanding of the pathophysiology of the host response, establishment of all‐quantitative severity scoring systems (e.g., APACHE and SOFA scores), and the definition of standard syndromes (e.g., sepsis and acute respiratory distress syndrome [ARDS]). In this era of acceleration, many randomized controlled trials (RCTs) have been conducted on drug therapy for critical illness, but results are sometimes inconsistent, and no standard treatments have been established. On the other hand, several therapies have been reported to have efficacy when targeted to specific patients. In a post hoc analysis of a double‐blind RCT (VANISH study) comparing the therapeutic effects of norepinephrine and vasopressin, hydrocortisone was associated with increased mortality in the molecular pathogenesis of septic shock, which shows non‐immunosuppressive forms.^4^ Early treatment with tranexamic acid was reported to be associated with improved outcomes in mild and moderate head trauma.^5^

Recent advances in measurement technology have enabled the precise evaluation of omics information, which refers to comprehensive biomolecular information (including comprehensive host DNA [genome], DNA modifications [epigenome], RNA [transcriptome], proteins [proteome], metabolite analysis [metabolome], and the coexistent microbiota in humans [microbiome]) **(**Table 1 **)**. Technological advancement has made it relatively easy to obtain comprehensive biological information using clinical specimens, thus enabling the evaluation of the detailed molecular pathology of patients with critical illness, opening this third era of precision medicine. In this era, the elucidation of novel molecular pathologies is expected to deepen the understanding of pathologies that could not be evaluated previously and to the realization of targeted and personalized treatment.

**TABLE 1:** List of Comprehensive Biomolecular Information (Omics Information).

Comprehensive biomolecular information(omics information)	Molecular information
Genome	The complete sequence information of DNA.
Epigenome	Acquired modifications made to the genome. Modifications include DNA methylation and methylation or acetylation of histones that store DNA.
Transcriptome	Comprehensive RNA information transcribed from the genome. Includes mRNAs that translate proteins and ncRNAs that do not translate.
Proteome	Comprehensive proteins expressed in the organism.
Metabolome	Comprehensive metabolites expressed in the organism
Microbiome	Microflora (bacteria, fungi, and viruses) present in the host.

## ADVANCES IN BIOINFORMATICS TECHNOLOGY FOR THE ANALYSIS OF COMPREHENSIVE BIOMOLECULAR INFORMATION

Over the past century, many experimental methods have been developed to analyze omics information. Advances in bioinformatics technology have enabled the analysis of omics information and multi‐omics analyses that combine omics information from different hierarchic levels and enabled a multifaceted understanding of disease states from a molecular biological perspective, leading to elucidation of the causal relationships of the cascades involved in immune responses and major cells and component molecules.^6^

During the 1990s and 2000s, significant improvements in sequencing technology, which is used to identify the primary structure in biomacromolecules including nucleic acids, and cost reductions of the sequencing technology led to a dramatic increase in data, termed biological big data. Moreover, the decline in measurement prices has facilitated clinical studies using sufficient numbers of clinical specimens.^7^ Bioinformatics has developed rapidly to cope with this explosion of biological big data, with biologists and computer scientists cooperating to develop new methods and tools. This has enabled the analysis, interpretation, and integration of vast amounts of genetic data and complex biological information, allowing researchers to understand biological processes and disease mechanisms on an unprecedented scale.^8^ Machine learning and artificial intelligence (AI) technologies have improved the automation of data analyses, pattern recognition, and predictive modeling, enabling researchers to gain biological insights more quickly and accurately.^9^

## NOVEL MOLECULAR DISEASE CLASSIFICATION BASED ON COMPREHENSIVE BIOMOLECULAR INFORMATION AND TARGETED PERSONALIZED MEDICINE

**FIGURE 3:**
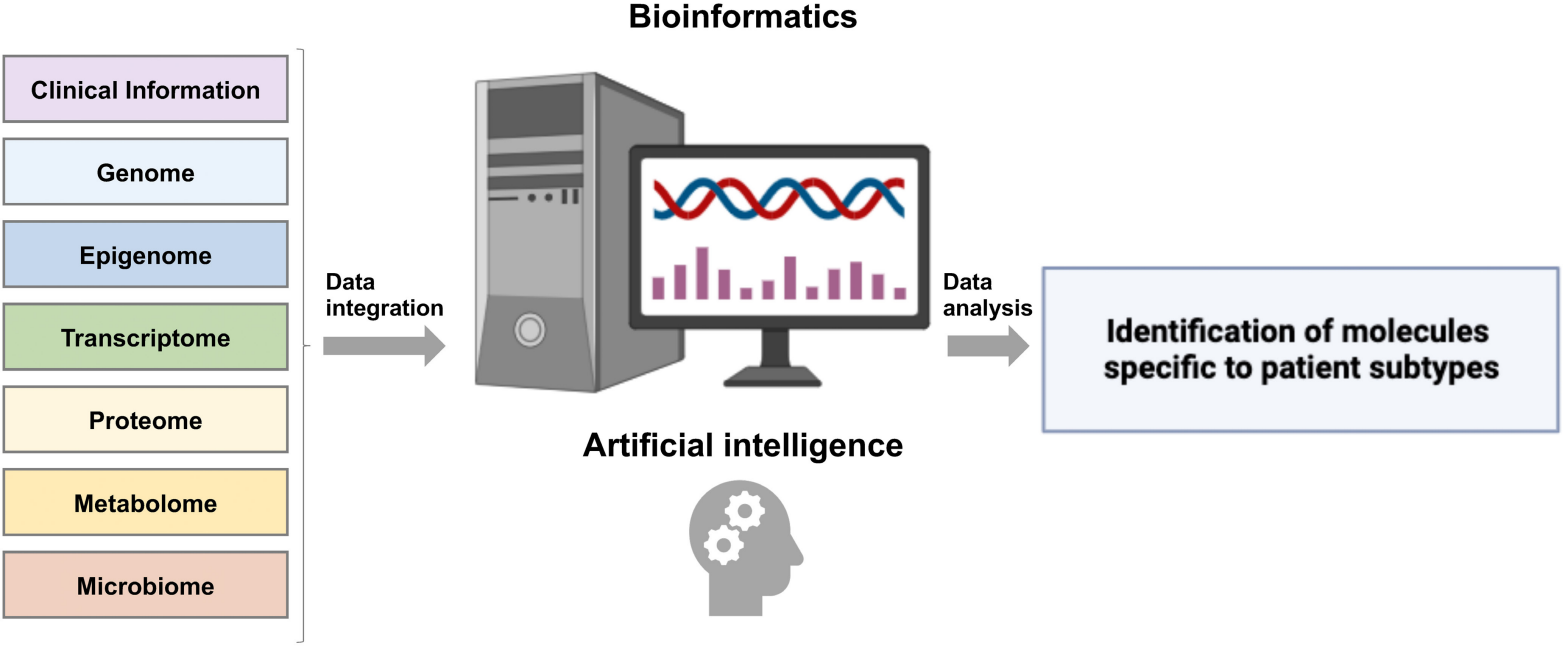
Bioinformatics analysis of comprehensive biomolecular information to elucidate novel molecular disease classifications. Clinical information and comprehensive biological information (omics information: genome, epigenome, transcriptome, proteome, metabolome, and microbiome) are integrated and analyzed using bioinformatics technology, including artificial intelligence (AI), to identify novel molecular disease classifications (i.e., subtypes) related to the prognosis.

Currently, critically ill patients are treated using a severity classification based on clinical symptoms, without considering the heterogeneity of host biological reactions. These patients are heterogeneous in terms of age, sex, race, living environment, causative organisms, and disease stage, and without sufficient patient selection, treatment may be ineffective. Recently, it was reported that the evaluation of comprehensive biomolecular information using clinical specimens can identify novel molecular pathology classifications (i.e., subtypes) associated with the prognosis and enable treatment targeting key molecules (Figure 3). Although the terminology defining novel molecular disease classifications is somewhat inconsistent, we define subtypes here to encompass novel molecular disease classifications based on clinical data, omics information, or data containing both.^3^

Despite numerous RCTs on patients with critical illness (sepsis, ARDS, etc.), supportive care has been the mainstay of treatment; therapeutic agents have been insufficient.^10^, ^11^ Recently, the importance of personalized medicine based on subtypes has been reported for patients with severe invasive disease.

Seymour et al. reported that four subtypes were identified using clinical information (e.g., demographic variables, vital signs, inflammation markers, and organ dysfunction markers) in a retrospective analysis of data sets from patients with sepsis. The subtypes correlated with host‐response patterns and clinical outcomes, suggesting that these phenotypes may help in understanding the heterogeneity of treatment effects.^12^

Scicluna et al. performed an unsupervised clustering analysis (i.e., grouping data into clusters based on similarities without using pre‐labeled categories or outcomes) using whole blood comprehensive RNA gene expression of sepsis patients to elucidate four subtype categories with different prognoses and distinct molecular pathologies that may require appropriate therapeutic intervention.^13^

Tachino et al. used machine learning with trauma patient data from the Japan Trauma Data Bank to identify poor prognostic subtypes, which were found to be associated with excessive inflammation and coagulation disorders,^14^ suggesting that early identification of poor prognostic subtypes from the initial medical data of trauma patients and preemptive treatment are possible.

Ebihara et al. identified major blood proteins based on a proteomic analysis of plasma from patients with severe COVID‐19 using the PEA method and reported that measurement of three proteins (WFDC2, CHI3L1, and KRT19) could identify the poor prognostic subtype, suggesting that measurement could predict severity of COVID‐19 at an early stage.^15^

The coronavirus disease (COVID‐19) pandemic caused by severe acute respiratory syndrome coronavirus 2 (SARS‐CoV‐2) infection paved the way for the possible establishment of effective treatments for the viral sepsis subtype. Specifically, in registry‐based RCTs such as the Recovery trial, patients with PCR‐diagnosed severe COVID‐19 were treated with corticosteroids, and interleukin‐6 inhibitors were shown to be effective,^16^ suggesting the possible effectiveness of targeted therapy in a homogeneous population if the heterogeneity of sepsis and ARDS can be subtyped.

## REVERSE TRANSLATIONAL RESEARCH TO APPROACH DRUG TARGET MOLECULES BASED ON COMPREHENSIVE BIOMOLECULAR INFORMATION USING CLINICAL SPECIMENS

**FIGURE 4:**
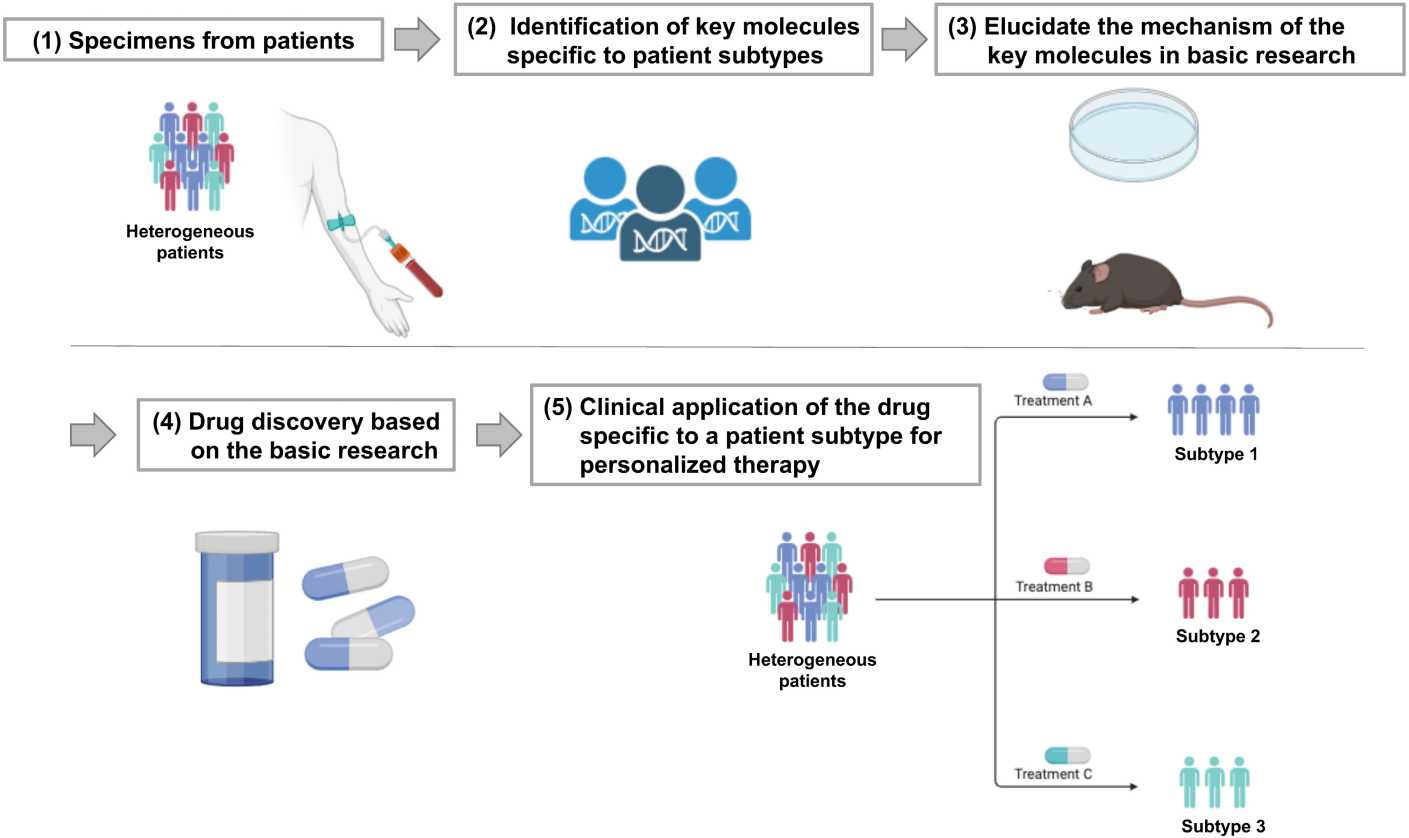
Reverse translational research to approach drug target molecules based on comprehensive biomolecular information using clinical specimens. Reverse translational research, also called systems biology, involves the following five processes: (1) Clinical research to obtain clinical specimens based on a hypothesis. (2) Analysis of comprehensive biomolecular information to identify key molecules directly related to clinical phenotypes, such as molecular pathological classifications (i.e., subtypes) with a poor prognosis. (3) Basic research on key molecules to verify their mechanisms using cell and animal (e.g., mouse) experiments. (4) Results of basic experiments lead to drug discovery targeting human molecules. (5) Targeting of subtypes for personalized therapy.

In this decade, new technologies have been developed to evaluate the human immune system with comprehensive biomolecular information. By measuring such information in clinical specimens, biomolecules that play roles in humans can be evaluated, enabling reverse translational studies in which findings from clinical studies are validated in basic research. The flow of data‐driven research based on comprehensive biomolecular information in humans is known as systems biology,^6^ in which a clear hypothesis is first formulated based on existing knowledge and clinical challenges. The experience and intuition of a specialist physician familiar with the clinical phenotype can be useful in developing such hypotheses. To test the hypothesis, relevant omics information is obtained in observational studies. Comprehensive biomolecular information is then analyzed in consultation with specialists as needed. This approach uses bioinformatics, including AI, to infer causal relationships within large‐scale data. For key molecules that may have a significant impact on the pathogenesis, the mechanism must be verified using cell or animal experiments. However, animal models do not necessarily reflect human immunology and should not be the sole means of verification. Recently, new methods of disease elucidation (e.g., human organoids and humanized chimeric mice) have emerged.^17^, ^18^ Research based on these methods will enable highly reproducible reverse translational studies based on clinical phenotypes and lead to drug discovery targeted to human molecules (Figure 4).

Measurement of comprehensive biomolecular information using clinical specimens is expected to lead to the complex identification of key molecules in humans and the elucidation of highly accurate pathological conditions and targeted treatment.^3^, ^19^ Although there is currently no large‐scale international project equivalent to the International Cancer Genome Consortium for critical illness, some large‐scale clinical studies on comprehensive biomolecular information have been reported. In sepsis, the application of a genome‐wide association study (GWAS) of genetic variation analysis, which uses information on genetic polymorphisms (i.e., variations in DNA sequences) scattered throughout the genome to search for genetic causes of disease, has been reported. Hernandez‐Beeftink et al. performed a GWAS meta‐analysis using whole blood from two sepsis groups and reported that the SAMD9 genetic single nucleotide polymorphism (rsID:rs34896991) was associated with survival.^20^ Scicluna et al. performed an unsupervised clustering analysis based on whole blood comprehensive RNA gene expression from three sepsis groups to elucidate four subtypes (MARS1‐4 groups) with different prognoses. The MARS1 group with the highest mortality could be identified by BPGM and TAP2 RNA gene expression with high predictive power (AUC = 0.99).^13^ In severe burns, Onishi et al. performed a proteomic analysis by mass spectrometry using plasma and identified three proteins (HBA1, TTR, and SERPINF2) strongly associated with the prognosis from 642 proteins.^21^ Additionally, a diverse multi‐omics blood atlas was created by the COVID‐19 Multi‐omics Blood Atlas (COMBAT) Consortium during the pandemic,^22^ which published comprehensive biomolecular information on COVID‐19. Also, an atlas of compiled databases was created with regard to COVID‐19.^23^, ^24^ Interestingly, comprehensive biomolecular information, including clinical information, was analyzed by AI to predict drugs that are expected to have a therapeutic effect on COVID‐19 (i.e., drug repositioning). Benevolent AI reported that baricitinib may be effective against COVID‐19 using an AI platform to analyze comprehensive biometric information, including clinical information.^25^ Baricitinib, a selective Janus kinase (JAK)1/JAK2 inhibitor, is used for autoimmune disease due to its anti‐inflammatory properties. Subsequently, baricitinib was found to inhibit human Numb‐related kinases (AAK1, BIKE, and GAK) involved in SARS‐CoV‐2 virus growth, and its efficacy was further validated in an RCT.^26^ Future evaluation of the roles of key molecules using drug repositioning may lead to new therapeutic targets.^27^

Technological innovations in the genetic domain have advanced therapeutic intervention methods, enabling intervention at the genetic level rather than only with conventional protein‐targeted and molecularly targeted drugs. Currently, nucleic acid medicine and gene therapy are two areas of drug discovery that require attention. Nucleic acid medicine refers to a type of treatment that uses nucleic acids to treat diseases by either silencing non‐beneficial genes or promoting beneficial gene expression. Nucleic acid medicine has been advancing, as exemplified by the practical application of mRNA vaccines (note: mRNA medicine is not nucleic acid medicine in the narrow sense but is included in nucleic acid medicine in the broad sense because it is made from nucleic acids).^28^, ^29^ Furthermore, nanomedicine technology, which involves using nanoparticles to deliver drugs, has evolved to transport nucleic acid drugs in a tissue‐specific manner.^30^ Effective tissue‐specific therapy facilitates targeted and precise treatment. These nucleic acid drugs have recently been approved or are in late‐stage development for several in vitro and in vivo gene therapies for infectious diseases, cancer, muscular dystrophy, retinal dystrophy, and other genetic disorders.^31^ Along with the elucidation of molecular pathogenesis, nucleic acid drugs are expected to be developed as tissue‐specific targeted therapeutics.^32^

Gene therapy is a therapeutic strategy that intervenes in the underlying cause of a particular disease by repairing defects in disease‐causing genes or introducing functional genes. Gene therapy is considered promising in the treatment of various diseases as well as hereditary diseases. Crisper/Cas9 genome editing technology is a precise tool that edits genes within organisms and is expected to lead to the development of gene therapies that control genes themselves. One gene therapy that has been clinically applied in oncology is CAR‐T therapy,^33^ in which T cells are genetically modified to produce chimeric antigen receptors (CARs) that bind specifically to cancer antigens, allowing T cells to recognize and target cancer cells more effectively. With the addition of gene editing, T‐cell therapies that can be implanted in any patient, independent of HLA type, have been proposed.^34^

With the further development of nucleic acid medicine and gene therapy, personalized treatment (i.e., N of 1 therapy) is expected.^35^

## CURRENT ISSUES AND FUTURE PROSPECTS

### Challenges and prospects for comprehensive biomolecular information measurement

Although it depends on the hierarchy of omics information, not all biomolecular information is currently covered. The advancement of measurement technology is expected to enable the identification of new molecules. The quality of comprehensive biomolecular information is greatly affected by the methods used to handle patient samples, measurements, and data standardization, even when samples are from the same patient. Therefore, when analyzing a specific disease group as a whole, it is extremely important to ensure uniformity in everything from sample handling to measurement conditions and standardization methods.

Furthermore, tissue specificity must be considered as the biological significance of information between different body fluids (e.g., blood or urine) or between different organs (e.g., kidney or liver) differs greatly. To properly interpret omics data, one must always be aware of the tissue or fluid from which data were obtained.

In critical illness, the patient's condition changes dynamically during the acute phase. Accordingly, the comprehensive biomolecular information also fluctuates significantly from time to time.^3^, ^36^ It is therefore useful to collect data over time that can closely track changes with disease progression. In reality, however, it is difficult to collect complete data over time in real time due to the burden of specimen collection, economic burden, and the time lag between the acquisition and the analysis of the measurement results.

### Challenges and prospects regarding bioinformatics analysis

With respect to data, the number of explanatory variables is overwhelmingly larger than the number of samples, and a bioinformatics analysis, which is different in quality from a clinical statistical analysis, is needed. Although bioinformatics analysis methods are progressing, there are several limitations.

In an omics analysis, RNA and protein expression levels within the same patient sample do not match perfectly. To address this, multidimensional analyses over time and from various tissues may be necessary.

Omics analyses require integration with clinical data (e.g., medical records, blood samples, imaging data, and physiological data). Non‐standardization of clinical data is also an issue. Efficient strategies for de‐identification, standardization, and sharing of clinical data to facilitate multicenter research remain a challenge. One potential solution is the development of a standardized format for the efficient exchange of clinical and physiological data: the HDF5‐based data exchange format allows for storage, compression, and real‐time streaming of multiparameter data. It can also integrate other large datasets (e.g., images and genomes).^37^

### Challenges and prospects for clinical research on novel molecular classification of pathological conditions

A polygenic risk score using a large number of genetic variants identified by GWAS has been developed to predict individual disease risk. It has been demonstrated that many diseases are derived from a complex association of polygenic mutations rather than mutations in a single gene.^38^ In addition to genetic complexity, one difficulty in a comprehensive bioinformatics analysis of critical illness is the complexity of the pathogenesis. Inflammation in sepsis is not localized inflammation, as occurs in cancer; it spills over to various tissues and organs throughout the body, including the vascular endothelium. Various cascades of immune, coagulation, regenerative, and metabolic reactions are associated with each type of inflammation, including cytokines with specific or common characteristics (Figure 2). Furthermore, host biological reactions differ depending on various factors, including patient background (age, sex, race, etc.), living environment, causative organisms, and disease stage.^3^, ^11^ To solve these issues, the development of a novel molecular pathology classification based on comprehensive biological information and treatment optimized for each pathology would be useful. In the future, it will be necessary to analyze molecular pathology continuously in real time using AI, including omics information, and to perform combined tissue‐specific targeted molecular therapy. This would enable precision therapies that comprehensively control inflammation while maintaining optimal homeostasis.^39^, ^40^

P4 medicine (predictive, personalized, preventive, participatory) has been proposed as a new approach in cancer care.^41^ Early intervention is obviously necessary even in critical illness, and P4 medicine may be useful. In addition to treating critical illness, preemptive medicine to prevent critical illness (e.g., diagnosis of susceptibility to critical illness and development of vaccines) is needed.

Various molecular pathology classification models and mortality prediction models have been reported. However, they have not been applied to clinical practice for critical illness because the analysis of molecular pathology classification in relation to prognosis is mainly based on observational studies and needs to be prospectively validated through RCT studies. One solution to this is a registry‐based RCT, in which data necessary for RCTs can be extracted from patient data enrolled in studies and analyzed in real time. During the COVID‐19 pandemic, REMAP‐CAP (Randomized Embedded Multifactorial Additive Platform for Community‐Acquired Pneumonia) was launched and used to provide rapid evidence.^42^ Additionally, the clinical application of real‐time, comprehensive biometric information has been suggested by the AI Clinician created by Komorowski et al.^43^ Providing real‐time, comprehensive biometric information from 5 ICUs and 128 hospitals using two ICU databases with 17,083 and 79,073 admissions, they developed AI Clinician to effectively apply molecular pathology classification models in real time; the decisions by AI exceeded those of human clinicians and favored long‐term survival. Another project to incorporate genetic information into clinical electronic medical records is the U.S. *e*MERGE project. *e*MERGE network research is currently underway at several medical institutions in the U.S. with the goal of combining medical records data with genetic information to provide physicians with the information they need to appropriately diagnose and treat individual patients.^44^

These clinical studies have suggested the importance of evaluating comprehensive outcomes, including activities of daily living. It is hoped that this will lead to patient‐centered care and the optimization of treatment strategies.^45^

### Challenges and prospects for basic research in reverse translational research

To link basic research to drug discovery, sufficient numbers of samples must be secured and the quality of comprehensive molecular information must be improved. The establishment of a platform for obtaining comprehensive molecular information is necessary.^46^ Specifically, the establishment of a biobank could ensure a uniform and diverse number of samples.^47^ Furthermore, aggregation of public data could lead to drug discovery by making data highly versatile.^48^ As a specific example, a blood atlas (COvid‐19 Multi‐omics Blood ATlas [COMBAT]) was created during the COVID‐19 pandemic to support future drug development.^22^ Moreover, an AI‐based analysis of aggregated and comprehensive molecular information of patients with COVID‐19 infection led to the selection and commercialization of baricitinib as a candidate drug.^25^ In the future, simulative drug discovery is expected to lead to drug discovery by reproducing in vivo molecular interrelationships in a virtual space.^49^

## CONCLUSIONS

In critically ill patients, the analysis of comprehensive biomolecular information reveals novel disease subtypes and drug targets that can enhance personalized medicine. This can promote personalized treatments in a diverse patient populations.

## FUNDING INFORMATIONS

None.

## CONFLICT OF INTEREST STATEMENT

Hisatake Matsumoto declares no conflicts of interest. Hiroshi Ogura is an Editorial Board member of AMS Journal and a co‐author of this article. To minimize bias, he was excluded from all editorial decision‐making related to the acceptance of this article for publication. Jun Oda is the Editor‐in‐Chief of the journal. He was excluded from the peer review process and all editorial decisions related to the acceptance and publication of this article. Peer review was handled independently by the AMS Journal editorial office and deputy EiC as editor to minimize bias.

## ETHICS STATEMENT

Approval of the research protocol: N/A.

Informed consent: N/A.

Registry and registration no. of the study/trial: N/A.

Animal studies: N/A.

## Data Availability

There is no data in the manuscript.
